# Improving grain yield prediction through fusion of multi-temporal spectral features and agronomic trait parameters derived from UAV imagery

**DOI:** 10.3389/fpls.2023.1217448

**Published:** 2023-10-16

**Authors:** Hongkui Zhou, Jianhua Yang, Weidong Lou, Li Sheng, Dong Li, Hao Hu

**Affiliations:** ^1^ Institute of Digital Agriculture, Zhejiang Academy of Agricultural Sciences, Hangzhou, China; ^2^ Academy of Eco-civilization Development for Jing-Jin-Ji Megalopolis, Tianjin Normal University, Tianjin, China

**Keywords:** yield prediction, agronomic trait, remote sensing, UAV, machine learning

## Abstract

Rapid and accurate prediction of crop yield is particularly important for ensuring national and regional food security and guiding the formulation of agricultural and rural development plans. Due to unmanned aerial vehicles’ ultra-high spatial resolution, low cost, and flexibility, they are widely used in field-scale crop yield prediction. Most current studies used the spectral features of crops, especially vegetation or color indices, to predict crop yield. Agronomic trait parameters have gradually attracted the attention of researchers for use in the yield prediction in recent years. In this study, the advantages of multispectral and RGB images were comprehensively used and combined with crop spectral features and agronomic trait parameters (i.e., canopy height, coverage, and volume) to predict the crop yield, and the effects of agronomic trait parameters on yield prediction were investigated. The results showed that compared with the yield prediction using spectral features, the addition of agronomic trait parameters effectively improved the yield prediction accuracy. The best feature combination was the canopy height (CH), fractional vegetation cover (FVC), normalized difference red-edge index (NDVI_RE), and enhanced vegetation index (EVI). The yield prediction error was 8.34%, with an R^2^ of 0.95. The prediction accuracies were notably greater in the stages of jointing, booting, heading, and early grain-filling compared to later stages of growth, with the heading stage displaying the highest accuracy in yield prediction. The prediction results based on the features of multiple growth stages were better than those based on a single stage. The yield prediction across different cultivars was weaker than that of the same cultivar. Nevertheless, the combination of agronomic trait parameters and spectral indices improved the prediction among cultivars to some extent.

## 1 Introduction

The growing global population has led to a rising demand for food. Increasing global climate change has caused frequent occurrences of natural disasters, posing a huge threat to agricultural production, and it has been demonstrated that climate change has a substantial effect on food security (Mora et al., 2018; Su et al., 2018; Misiou and Koutsoumanis, 2022). Comprehensive, timely, and accurate grain yield prediction of major crops is also of great significance for optimizing the structure of the agricultural industry and formulating rural development plans. Therefore, whether in the context of current climate change or macro policies, it is quite necessary to quickly and accurately estimate crop yields to ensure food security and agricultural and rural development.

Traditionally, crop yield prediction has mainly relied on field surveys, which require much time, people, and resources. Currently, crop yield prediction methods include statistical regression models, crop model simulations, and remote sensing (RS)-based models. The deficiency of statistical regression models is that the yield prediction accuracy is related to the crop cultivars, region, and growth period, and the models are not universal (Fang et al., 2011; Huang et al., 2015). The main superiority of the crop model simulation method is that it can mechanically simulate the entire process of crop growth and biomass accumulation. However, the accuracy of the production simulation depends on the model structure and the accuracy of the model parameters, and there are many parameters required (Asseng et al., 2013; Dong et al., 2020). Therefore, it is still challenging to accurately estimate production on a large scale. RS technology has developed rapidly in recent years, and it has been widely used in crop yield prediction due to its advantages of large coverage area, low cost, and high efficiency (Sagan et al., 2021).

Currently, many studies have used satellite RS images to predict the crop yield and have achieved a good estimation accuracy. These studies involved a variety of methods (e.g., statistical regression, machine learning, and data assimilation), various crop types (e.g., rice, wheat, cotton, and potatoes), and different RS data (from low to high resolution, from multispectral (MS) to hyperspectral (HS) bands) (Lobell et al., 2015; Lambert et al., 2018; Yang et al., 2019; Filippi et al., 2019; Sakamoto, 2020; van Klompenburg et al., 2020; Weiss et al., 2020; Cao et al., 2021; Sagan et al., 2021; Jeong et al., 2022). With the continuous development of precision agriculture, the requirements for crop yield prediction in terms of spatial resolution and accuracy have increased (Maes and Steppe, 2019). Satellite imagery still has the problem of low spatial resolution for farmland with a small area and complex terrain. In addition, it is easily affected by rainy weather, resulting in poor image continuity. Therefore, due to the advantages of ultra-high spatial resolution and flexibility, unmanned aerial vehicle (UAV) RS platforms have been significantly improved in many agricultural applications, such as crop yield prediction, field management, crop phenology identification, and chlorophyll estimation in recent years (Maresma et al., 2016; Maes and Steppe, 2019; Li et al., 2021; Guo et al., 2022; Tanabe et al., 2023).

The main idea of many existing studies is to use digital cameras and MS and/or HS sensors carried by UAVs to obtain or estimate various parameters related to the crop yield and then to apply statistical or machine learning techniques to predict the crop yield (van Klompenburg et al., 2020; Sagan et al., 2021). Nonetheless, the accuracy and robustness of the crop yield prediction still need to be further improved. The accuracy and robustness can be further improved by (1) optimizing the feature parameter space of the crop yield prediction and selecting more suitable features; (2) improving crop yield prediction algorithms; and (3) combining other yield prediction methods (e.g., crop model simulations). This study mainly focused on the first method. Through a review of the existing literature, it was found that most studies have used the spectral features of crops, especially vegetation indices or color indices to predict crop yields. Vegetation indices exhibit a strong correlation with crop growth and development when the coverage is low. However, they are prone to saturation when the canopy of the plant is closed, at which time they become less sensitive to the plant growth. In addition, the vertical growth information which is strongly linked to the formation of crop biomass and yield, poses a challenge for vegetation indices to detect accurately during the middle and later stages of crop growth (Yue and Tian, 2020). Therefore, in addition to spectral features, it is necessary to improve the feature space for yield prediction and to select optimal and available agronomic RS features that are closely related to the yield formation.

Agronomic trait parameters are closely linked with crop growth and yield formation, so they are considered to have great potential for improving the yield prediction capability. Many agronomic trait parameters involve all aspects of the crop growth process, and they can also be acquired through RS techniques. The agronomic trait parameters in this study specifically refer to those obtained using RS techniques. Choosing parameters related to crop yield and relatively independent of crop growth is an important principle for feature selection. Many RS-based agronomic biochemical/biophysical parameters (e.g., the chlorophyll content, nitrogen content, and leaf area index) are usually obtained using the relationship with vegetation indices, and hence, they are autocorrelated with the spectral features. The fractional vegetation cover (FVC) is crucial parameter that describes the spatial pattern of vegetation types, and it is closely relevant to the crop planting density, growth stage, and health status (Gao et al., 2020). The canopy height (CH) and canopy volume (VOL) can reflect the vertical growth of crops and can characterize the crop structure information (Maimaitijiang et al., 2019; Zhang et al., 2021; Shu et al., 2022). The three indicators mentioned above are all agronomic structural trait parameters that are closely related to the yield, and all three can be obtained using a UAV. In addition, compared with spectral or color information, they are relatively independent data sources. The FVC can be calculated using the image classification method, while the CH and VOL are extracted from dense photogrammetric point cloud information obtained by a UAV equipped with a high-definition camera. In addition, the texture is also a frequently used RS feature that can provide insight into the spatial variations within the vegetation canopy to a certain extent. Currently, the abovementioned metrics have been applied for predicting nitrogen content, crop biomass, and crop yield. Nevertheless, there is an ongoing need for further validation on how to better integrate multi-temporal spectral features with agronomic trait parameters to enhance the accuracy of yield predictions. Additionally, the adaptability of the constructed models across different crop cultivars still requires further explored.

Machine learning has become a key approach to predict crop yield using UAV-based RS data (Shahhosseini et al., 2020; van Klompenburg et al., 2020; Wang et al., 2021; Xu et al., 2021). The random forest (RF) is a widely used machine learning algorithm with many advantages (Breiman, 2001; Li et al., 2020; He et al., 2021). Firstly, it is an ensemble learning algorithm that achieves predictions by constructing multiple decision trees, each with a degree of independence. As a result, it exhibits robustness to noise, outliers, and missing values, making it highly reliable. Secondly, RF introduces a bootstrap sampling mechanism, which enhances the model’s generalization ability while mitigating the risk of overfitting. Furthermore, it is relatively easy to use and does not require extensive hyperparameter tuning. Importantly, RF has been proven to perform well in many studies (Li et al., 2020; Marques Ramos et al., 2020; van Klompenburg et al., 2020; Wan et al., 2020). Therefore, we used the RF algorithm as the core algorithm and combined it with spectral features, texture features, and agronomic trait parameters based on UAV images to predict the crop yield. The specific research goals of this study were (1) to predict the crop yield and compare the performances of the spectral, texture, and agronomic trait parameters; (2) to evaluate the impacts of the parameters in the different growth periods on the yield prediction results; and (3) to investigate the robustness of models of different cultivars and to evaluate whether the incorporation of agronomic parameters can enhance the predictive capacity of the crop yield model for various cultivars. This study focuses on wheat as its research crop, aiming to estimate its yield. It should be noted that in this context, ‘yield’ specifically refers to grain yield rather than biomass yield.

## 2 Materials and methods

### 2.1 Experimental design

**Figure 1:**
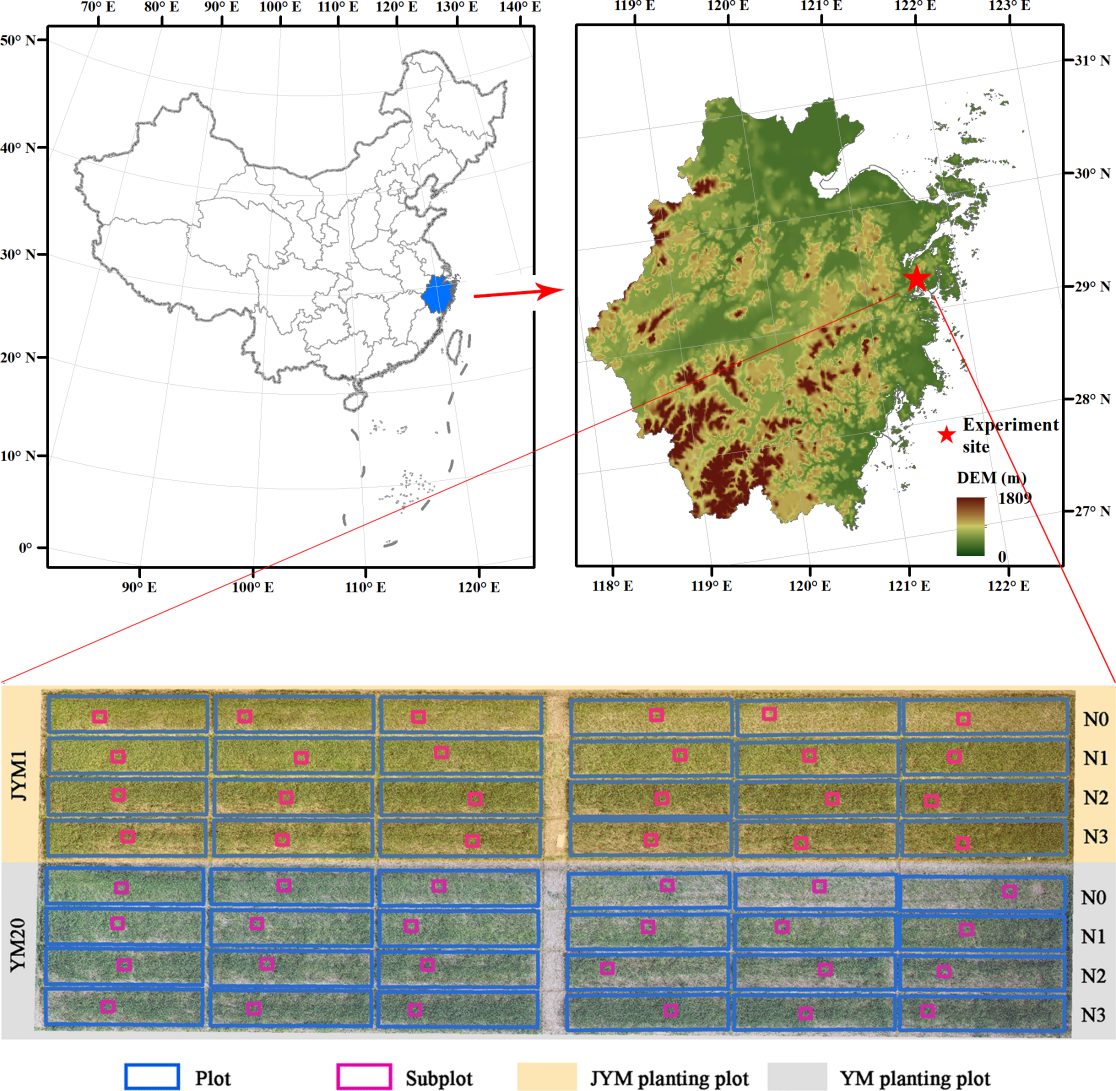
Location of the study area and experimental design.

The study was conducted at the experimental site situated in Ningbo City, Zhejiang Province, with geographic coordinates of 29°18′N and 121°34′E. The study area has a subtropical monsoon climate characterized by clear seasonal variations. The average temperatures in summer and winter are approximately 27°C and 6°C, respectively, resulting in an annual average temperature of approximately 16°C. The average annual rainfall is approximately 1700 mm. In this study, winter wheat was selected as the research crop, which is one of the most important crops in the study area. The experimental period was the 2019–2020 winter wheat growing season (planting in November 2019 to harvest in May 2020). The experimental design is shown in **Figure 1**. Two main wheat cultivars (JYM 1 and YM 20) were used. For each cultivar, four nitrogen fertilizer treatments and six replicates were set, i.e., 24 plots for each cultivar. There were 48 plots (3 × 13.7 m) in the entire experiment, and each plot had a subplot (1 × 1 m). The nitrogen fertilizer treatments were 0 (N0), 90 kg/ha (N1), 180 kg/ha (N2), and 270 kg/ha (N3). The application rates of the phosphate fertilizer and potash fertilizer were the same in each plot. The amount of phosphate fertilizer was 75 kg/ha, and the amount of potash fertilizer was 120 kg/ha. Nitrogen fertilizer was applied twice: 40% of the total amount was applied during the sowing, and the remaining 60% was applied in the jointing stage. The phosphate fertilizer and potash fertilizer were applied once during the sowing.

### 2.2 Data collection

#### 2.2.1 Collection and processing of UAV images

In this study, two UAVs (Phantom 4 RTK, SZ DJI Technology Co., Ltd., China), one equipped with a red-green-blue (RGB) camera and the other equipped with an MS camera, were employed to capture RGB and MS images during the winter wheat growing season. The basic parameters of the UAV and onboard sensors are described in **Table 1**.

**Table 1:** Parameters of the UAV and onboard RGB and MS sensors.

Sensor	Band	Spectral range (nm)	Resolution (pixels)	Field of view (°)	Positioning accuracy (cm)	Ground resolution at 100m height (cm)
RGB camera	RGB	/	5472×3648	84	Horizontal: 1 Vertical: 1.5	2.74
MS camera	Blue (B)	450 ± 16	1600×1300	62.7	Horizontal: 1 Vertical: 1.5	5.3
	Green (G)	560 ± 16				
	Red (R)	650 ± 16				
	Red Edge (RE)	730 ± 16				
	Near Infrared (NIR)	840 ± 26				

Seven UAV flight missions were conducted during the critical growth stage of the winter wheat. The flight dates and corresponding growth stages are listed in **Table 2**. Under clear weather conditions, the RGB and MS images were collected between 10:00 and 14:00 local time. The flight height of the UAV was 30 m; the forward and side overlap ratios were set to 80% and 70%, respectively.

**Table 2:** Seven UAV fight dates and corresponding wheat growth stages.

Flight date	Growth stage	Abbr.
Mar 16, 2020	Jointing	JS
Mar 26, 2020	Booting	BS
Apr 2, 2020	Heading	HS
Apr 15, 2020	Initial filling	IFS
Apr 24, 2020	Middle filling	MFS
Apr 29, 2020	Late filling	LFS
May 12, 2020	Maturity	MS

After obtaining the aerial photos of the study area, the photos were preprocessed, comprising two major procedures: (1) image mosaicking in a single period and (2) geometric correction between the mosaicked images in different periods. The image mosaicking included the following steps: image registration of each band, vignetting correction, distortion calibration, and radiation correction. The above image mosaicking steps were all performed using the DJI Terra software (SZ DJI Technology Co., Ltd., China) designed for DJI UAVs. For radiometric calibration, three calibration whiteboards with reflectance values of 25%, 50%, and 75% were placed beneath the flight path of the UAV, and collected in the multispectral sensor. In DJI Terra V3.5.5, the raw image’s DN (Digital Number) values were transformed into surface reflectance using a linear correction method (Xia et al., 2022). The corrected images were mosaicked into multi-temporal RGB and reflectance images of the study area. Then, all of the mosaicked images for the different periods were resampled into images with a resolution of 2 cm. Geometric registration was performed on these resampled images to ensure that the pixel positions of the images in all of the periods corresponded to each other. This process was completed using the ArcGIS software (Esri, Inc., Redlands, CA, USA).

#### 2.2.2 Crop yield measurements

After the wheat matured, the 48 plots and 48 subplots were harvested to obtain yield measurements. The manual harvesting method was used to reduce the error of the yield measurements. The harvested wheat was threshed in the laboratory, and the grain water content was measured. The formula used to calculate the wheat yield is as follows:


(1) $$Y_{m}=10000*\text{G}÷\text{A}\times (1-\text{C})÷(1-13\%)$$

where $Y_{m}$ is the wheat yield (kg/ha); G is the weight of the harvested wheat seeds in each plot (kg); A is the plot area (m^2^); C is the grain moisture content (%); and 13% is the wheat standard moisture content (Xin et al., 2008).

### 2.3 Yield prediction model development

**Figure 2:**
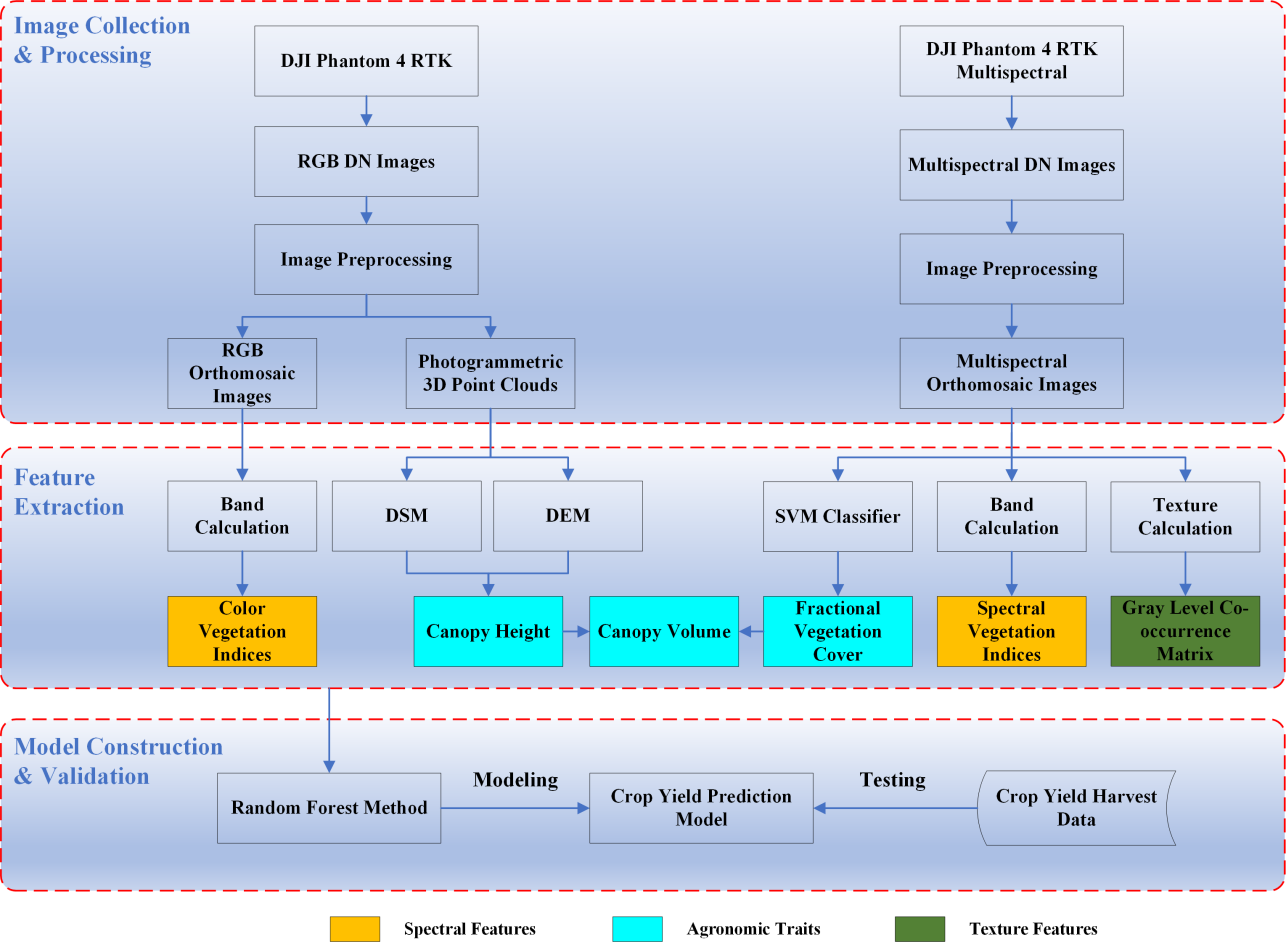
Workflow of the development of the yield prediction model.

**Figure 2** shows the workflow of the development of the crop yield prediction model in this study, comprising three parts: image collection and processing, feature extraction, and model construction and validation. Section 2.2 introduced the image acquisition and preprocessing. This section mainly describes the image feature extraction and model building.

#### 2.3.1 Feature extraction

##### 2.3.1.1 Spectral features

The main variables used to represent the spectral features in this study were the original values (i.e., the band reflectance and RGB values) of the UAV MS and RGB images and the vegetation/color indices (**Table 3**) calculated based on the original values.

**Table 3:** Summary of the vegetation/color indices used in this study.

Sensors	Vegetation/color indices	Abbreviations	Equations	References
MS	Normalized difference vegetation index	NDVI	(NIR−R)/(NIR+R)	Rouse et al., 1974
	Green normalized difference vegetation index	GNDVI	(NIR−G)/(NIR+G)	Gitelson et al., 2003
	Enhanced vegetation index	EVI	2.5×(NIR−R)/(NIR+6×R−7.5×B+1)	Huete et al., 2002
	Enhanced vegetation index without a blue band	EVI2	2.5×(NIR−R)/(NIR+2.4×R+1)	Jiang et al., 2008
	Modified triangular vegetation index	MTVI2	$1.5*\left[1.2*\left(\text{NIR}-\text{G}\right)-2.5*\left(\text{R}-\text{G}\right)\right]/\sqrt{{\left(2\text{*NIR}+1\right)}^{2}-\left(6*\text{NIR}-5\sqrt{R}\right)-0.5}$	Haboudane, 2004
	Soil-adjusted vegetation index	SAVI	1.5×(NIR−R)/(NIR−R+0.5)	Huete, 1988
	Normalized difference red-edge index	NDVI_RE	(NIR−RE)/(NIR+RE)	Gitelson and Merzlyak, 1994
	Modified simple ratio red-edge index	MSR_RE	$\left(\text{NIR}/\text{RE}-1\right)/\sqrt{\text{NIR}/\text{RE}+1}$	Wu et al., 2008
	Red-edge chlorophyll index	CI_RE	NIR/RE−1	Gitelson et al., 2003
RGB	Normalized difference index	NDI	(g−r)/(g+r)	Woebbecke et al., 1995
	Excess green index	ExG	2×g−r−b	Woebbecke et al., 1995
	Excess red index	ExR	1.4×r−g	Meyer and Neto, 2008
	Excess green minus excess red index	ExGR	3×g−2.4×r−b	Meyer and Neto, 2008
	Visible atmospherically resistant index	VARI	(g−r)/(g+r−b)	Gitelson et al., 2002
	Green leaf index	GLI	(2×g−b−r)/(2×g+b+r)	Louhaichi et al., 2001
	Normalized difference yellowness index	NDYI	(g−b)/(g+b)	Sulik and Long, 2016

R, G, B, Nir, and RE denote the reflectance in the red, green, blue, near-infrared, and red-edge bands for the MS images, respectively; and r, g, and b are the normalized DNs of the red, green, and blue channels for the RGB images, respectively.

##### 2.3.1.2 Image textures

The gray level co-occurrence matrix (GLCM) is a frequently utilized and widely adopted method for calculating image texture features, and it was used to represent the image texture feature in this study. The GLCM consists of eight features: the mean (MEA), variance (VAR), homogeneity (HOM), contrast (CON), dissimilarity (DIS), entropy (ENT), second moment (SEM), and correlation (COR). The details of the specific calculation methods have been described by Haralick et al. (1973). In this study, a moving window with size of 3×3 and a co-occurrence shift of 1 pixel were utilized for texture calculations. The ENVI software (L3Harris Technologies, Inc., Boulder, CO, USA) was used to calculate the GLCM features for seven temporal MS images, and a total of 280 texture features were generated.

##### 2.3.1.3 Agronomic traits

Many parameters characterize the growth and development of crops, including biochemical, biophysical, and structural parameters. In this study, three RS-based, available, and independently sourced traits were selected for use in the crop yield prediction.

###### 2.3.1.3.1 Canopy height

A digital surface model (DSM) can be obtained using the photogrammetric 3-D point clouds from the UAV RGB images (Colomina and Molina, 2014; Maimaitijiang et al., 2017). Therefore, a DSM of the crop canopy was generated from the UAV RGB images during the crop growth and development stages. Similarly, a digital elevation model (DEM) of the bare soil surfaces in the study area was obtained from the UAV flight before wheat germination. The DEM was subtracted from the canopy DSM to obtain the wheat CH [Eq. (2)].


(2) CH=DSM−DEM

The specific processes were as follows. First, the DEM and canopy DSMs for the different periods were obtained using the DJI Terra software. Second, it was necessary to ensure that the DSM and DEM had the same resolution, and the pixels corresponded to each other. Finally, the CH was calculated pixel by pixel using Eq. (2).

###### 2.3.1.3.2 Fractional vegetation cover

The FVC is a crucial parameter that describes the spatial pattern of the vegetation types and can serve as an indicator for monitoring vegetation health (Yan et al., 2019; Gao et al., 2020). There are currently many RS methods for estimating the FVC (Gao et al., 2020). In this study, the supervised classification method was used to distinguish between the soil and crop information based on the UAV MS images. Specifically, the support vector machine (SVM) classifier was selected as the supervised classification method to identify crop pixels. Previous studies have shown that the SVM has a higher classification accuracy in the case of relatively limited samples (Mountrakis et al., 2011; Maimaitijiang et al., 2020; Wan et al., 2020). Subsequently, the FVC was calculated using Eq. (3).


(3) $$\text{FVC}=\frac{\text{c}}{\text{n}}\times 100$$

where c is the number of crop pixels in the plot, and n is the total number of all pixels in the plot.

###### 2.3.1.3.3 Canopy volume

The canopy volume (VOL) reflects the three-dimensional structure of the crops during the growth and development stages. Existing studies have used it in crop biomass estimation (Walter et al., 2018; Maimaitijiang et al., 2019) and have achieved good estimation results. In this study, we attempted to use the VOL as one of the features for crop yield estimation. The formula for calculating the VOL is as follows:


(4) $$\text{VOL}=\sum_{i=1}^{c}(A_{i}\times CH_{i})$$

where VOL is the canopy volume; c is the number of crop pixels in the plot; $A_{i}$ is the area of the pixel *i*; and $CH_{i}$ is the crop height in pixel *i*.

#### 2.3.2 Yield prediction model

The RF algorithm (Breiman, 2001) was used to construct the models for wheat yield prediction. The RF belongs to the category of ensemble learning algorithms, and uses the bootstrap sampling method to build a large number of independent decision trees to implement classification and regression tasks. The RF is insensitive to collinearity between variables, can effectively reduce the problem of overfitting, and has been proven to perform well in many studies (e.g., crop parameters, biomass, yield estimation, and image classification) (Li et al., 2020; Wan et al., 2020; He et al., 2021). In this study, the number of decision trees, ntree, was set to 500, and the default values were used for the rest of the RF parameters. There was a total of 96 plot samples (including subplots) in this study, and 2/3 of the data were selected for model training, while the remaining 1/3 of the data were independently employed for model testing.

### 2.4 Evaluation metrics

The evaluation metrics included Pearson’s correlation coefficient (*R*), coefficient of determination (*R^2^*), root mean square error (*RMSE*), and relative root mean square error (*RRMSE*). The *R* value was used to analyze the relationship between each feature and the crop yield, and the *R^2^*, *RMSE*, and *RRMSE* values were used to measure the accuracy and error of the yield prediction model. The calculation formulas of the statistical analysis indicators are as follows:


(5) $$R=\frac{\sum_{i=1}^{n}\left(x_{i}-\bar{x}\right)\left(y_{i}-\bar{y}\right)}{\sqrt{\sum_{i=1}^{n}{\left(x_{i}-\bar{x}\right)}^{2}\sum_{i=1}^{n}{\left(y_{i}-\bar{y}\right)}^{2}}}$$

(6) $$RMSE==\sqrt{\frac{1}{\text{n}}\sum_{i=1}^{\text{n}}{\left(x_{i}-y_{i}\right)}^{2}}$$

(7) $$RRMSE=\frac{RMSE}{\bar{x}}\times 100\%$$

where x and y are the observed and predicted variables, respectively; $\bar{x}$ and $\bar{y}$ are the average values; and n is the number of observations.

## 3 Results

### 3.1 Correlations between model features and crop yield

**Figure 3:**
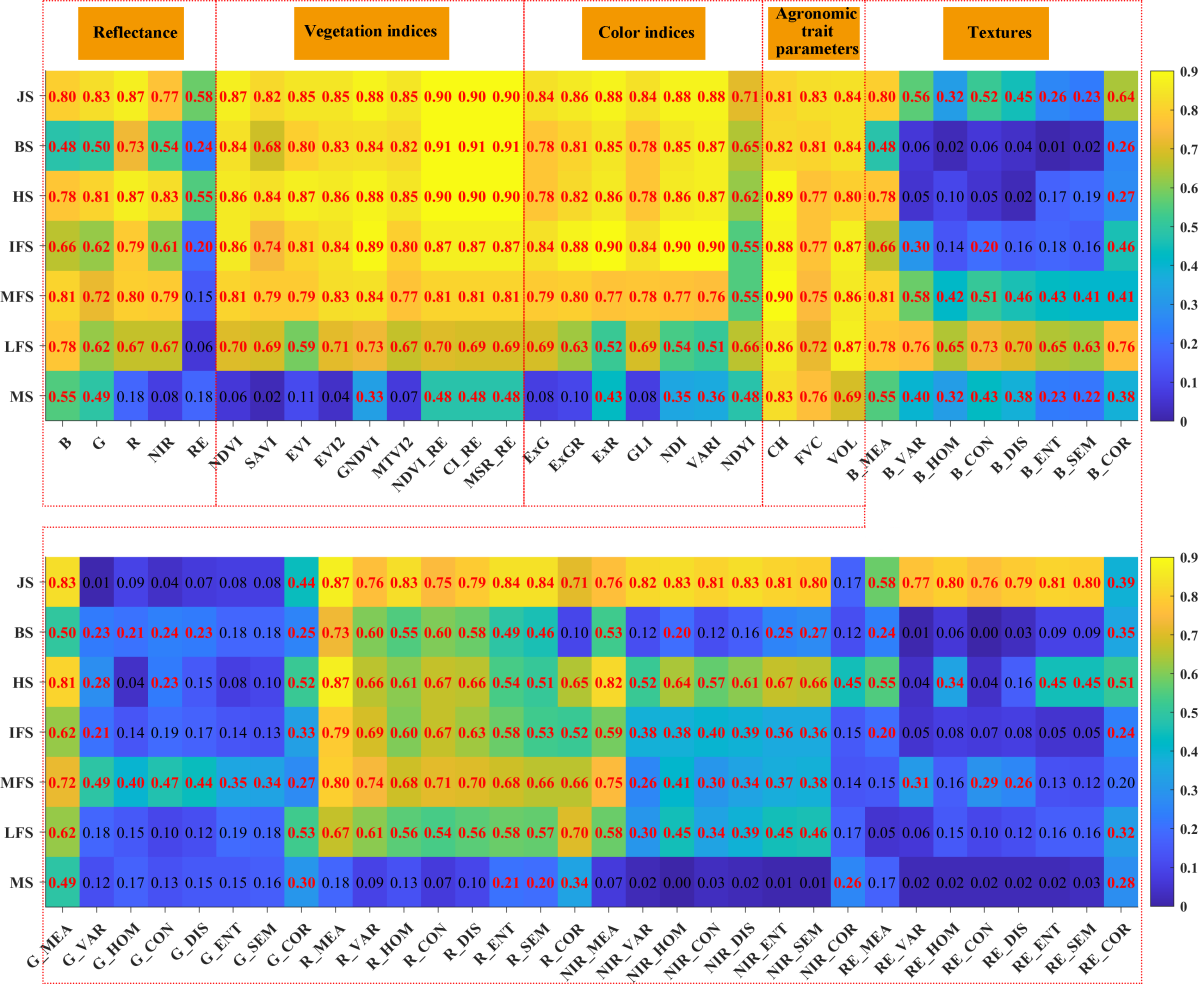
Correlations between various features (i.e., reflectance, vegetation/color indices, agronomic trait parameters and textures) and crop yield. The red font represents that the correlation is significant at the 0.01 level.

**Figure 4:**
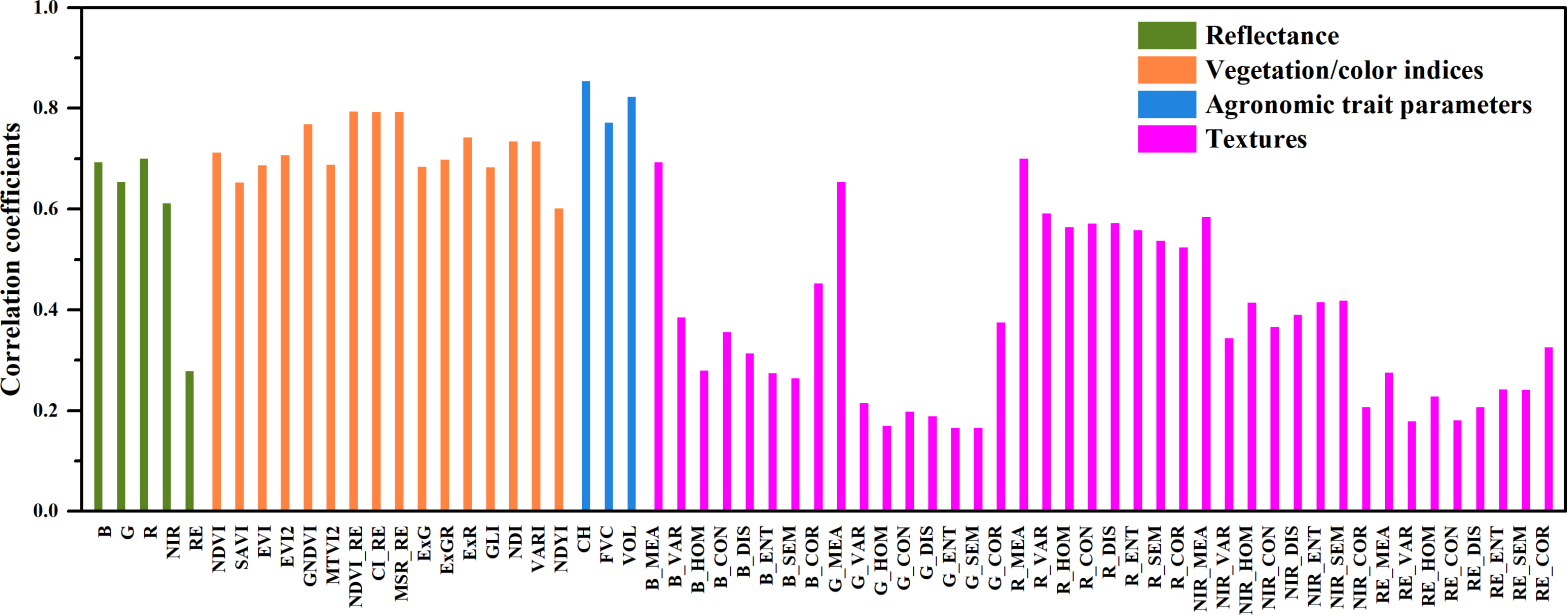
The average values of the correlation coefficients between the yield and remote sensing features in the different growth stages.

Correlation analysis was conducted to investigate the relationships between the model feature parameters and the crop yield so as to better screen the optimal features for crop yield prediction. **Figures 3** and **4** show the correlations between the features of four categories of features (reflectance, vegetation/color indices, agronomic trait parameters, and textures) and the crop yield, as well as the average values of the correlation coefficients during the different growth periods. In general, among the four categories of features, the agronomic traits have strong correlations with the crop yield, followed by the vegetation/color indices and reflectance, and the texture features exhibit relatively weak correlations. The agronomic trait parameters (FVC, CH, and VOL) have good correlations with the crop yield during each growth stage. They all pass the 0.01 significance level test, and their average correlation coefficients are 0.77, 0.85, and 0.82, respectively (**Figure 4**). For the vegetation indices, the red-edge vegetation indices (REVIs) have better correlations with the crop yield, and the correlations in the jointing, booting, and heading stages are > 0.9. For the color indices, the NDYI performs better, and the relationships between the other color indices and the crop yield are weaker. For the texture features, except for the red band features, most of the other features exhibit weak correlations.

### 3.2 Yield prediction using a single feature

**Figure 5:**
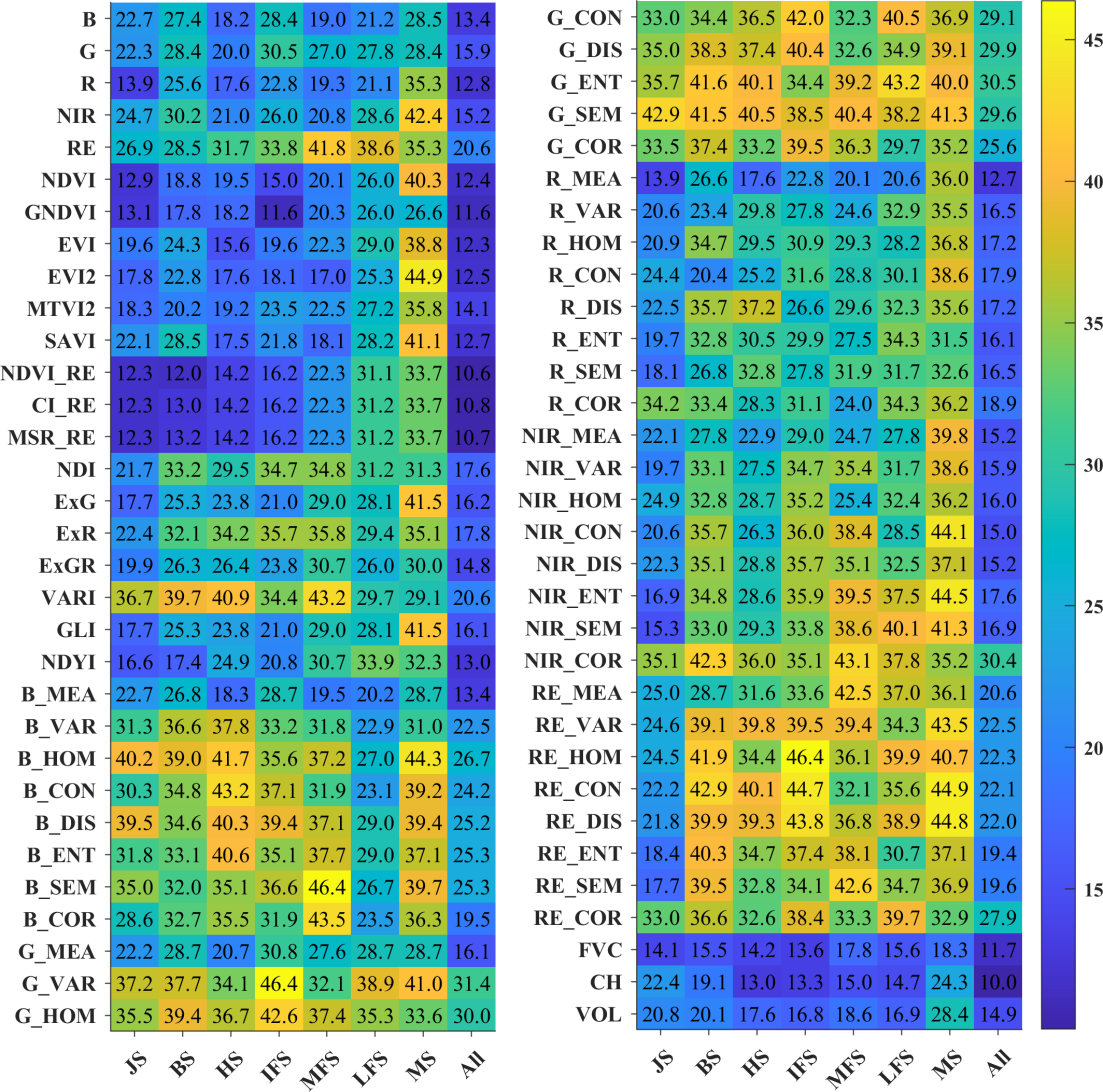
The *RRMSEs* (%) of the yield predicted using the remote sensing features of the different growth stages.

An RF-based yield estimation model was constructed using a single feature, and the yield was predicted using the feature parameters in the different growth stages and during the entire growth period. **Figure 5** shows the error (*RRMSE*) of the yield prediction result. There are great differences in the yield accuracy obtained using the features in the different growth stages and the different categories (reflectance, vegetation indices, textures, and agronomic trait parameters). Specifically, using the features of the entire growth stage leads to significantly smaller yield errors than using the features of a single growth stage. The errors of the yield prediction obtained using the features of the entire growth stage are 10–30.4%, with an average value of 18.7%. Furthermore, the errors of the yield prediction obtained using a single feature are 11.6–46.4%, with an average value of 30.1%.

**Figure 6:**
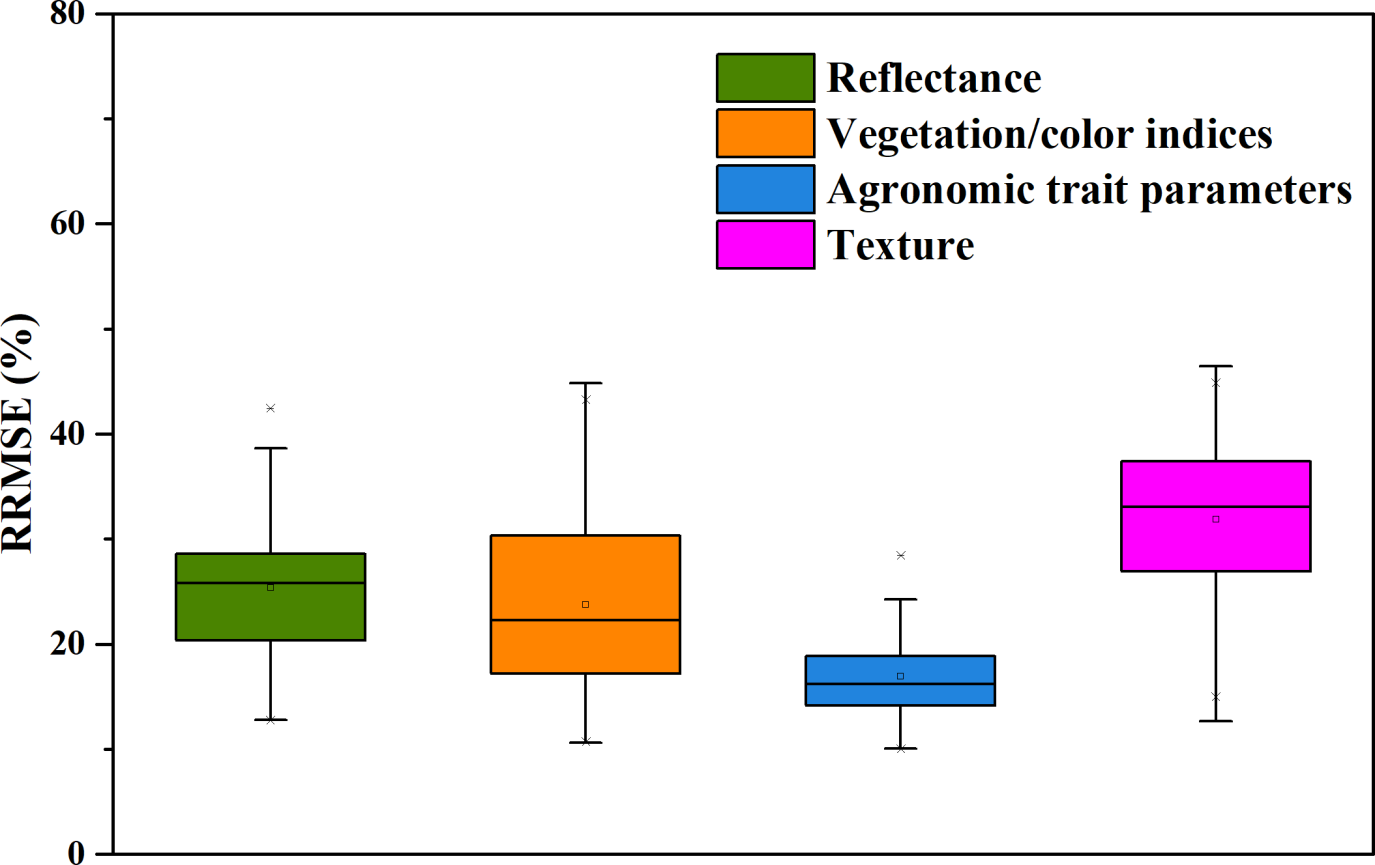
Box plots of the errors of the predicted yield obtained using the different categories of feature parameters.

In addition, the performances of the different categories of feature variables in the yield prediction were compared. **Figure 6** presents a box plot of the error of the yield prediction of the feature variables of each category (reflectance, vegetation indices, textures, and agronomic trait parameters). The results show that similar to the correlation analysis results, the average error of the yield prediction obtained using the agronomic trait parameters is the smallest, followed by that obtained using the vegetation indices and reflectance, and the relative error of the yield prediction obtained using the texture features is the largest. Overall, the agronomic trait parameters perform the best in the yield prediction, and the error of the yield prediction obtained using the plant height parameter for the entire growth period is the smallest, with an *RRMSE* of 10%.

### 3.3 Yield prediction using combinations of multiple features

In Sections 3.1 and 3.2, it was found that the different categories of feature parameters have differences in predicting the crop yield. The agronomic trait parameters and vegetation/color indices perform better. Therefore, multiple features of agronomic trait parameters and vegetation/color indices were integrated to determine the best combination of yield prediction features. To compare the vegetation indices with different construction principles, they were subdivided into the commonly used vegetation indices of the near-infrared and visible light bands (ComVIs), the red-edge vegetation indices (REVIs), and the color indices (CIs). **Table 4** shows the error statistics of the optimal yield prediction results for different feature combinations using all of the growth stage data.

**Table 4:** The error statistics of the yield prediction results based on various feature combinations.

Types	Feature variables	Number of variables	Number of combinations	Best combination	*RRMSE* (%)	*R^2^*
ComVIs	NDVI, GNDVI, EVI, EVI2, MTVI2, SAVI	6	63	GNDVI, SAVI	10.47	0.91
REVIs	NDVI_RE, MSR_RE, CI_RE	3	7	NDVI_RE	10.57	0.91
CIs	NDI, ExG, ExR, ExGR, VARI, GLI, NDYI	7	127	ExG, VARI	15.79	0.78
AgTP	CH, FVC, VOL	3	7	CH, FVC	8.93	0.94
ComVIs+ REVIs+CIs	NDVI, GNDVI, EVI, EVI2, MTVI2, SAVI, NDVI_RE, MSR_RE, CI_RE, NDI, ExG, ExR, ExGR, VARI, GLI, NDYI	16	65535	NDVI_RE, MSR_RE, EVI, SAVI	9.88	0.92
ComVIs+AgTP	NDVI, GNDVI, EVI, EVI2, MTVI2, SAVI, CH, FVC, VOL	9	511	CH, FVC, SAVI, GNDVI	8.85	0.94
REVIs+AgTP	NDVI_RE, MSR_RE, CI_RE, CH, FVC, VOL	6	63	CH, FVC, NDVI_RE	8.36	0.94
CIs+AgTP	NDI, ExG, ExR, ExGR, VARI, GLI, NDYI, CH, FVC, VOL	10	1023	CH, FVC, VARI	8.52	0.95
ComVIs+REVIs+ CIs+AgTP	NDVI, GNDVI, EVI, EVI2, MTVI2, SAVI, NDVI_RE, MSR_RE, CI_RE, NDI, ExG, ExR, ExGR, VARI, GLI, NDYI, CH, FVC, VOL	19	524287	CH, FVC, NDVI_RE, EVI	8.34	0.95

ComVIs, commonly used vegetation indices with near-infrared and visible light bands; REVIs, red-edge vegetation indices; CIs, color indices; AgTP, agronomic trait parameters.

The results show that the minimum *RRMSE* of the yield prediction, based on the vegetation indices, reduced from 11.6% for a single feature (GNDVI) (**Figure 5**) to 9.88% for multivariate combinations (NDVI_RE, MSR_RE, EVI, and SAVI) (**Table 4**). There are also differences in the yield prediction accuracy based on the combination of vegetation indices, and the estimation accuracy based on the ComVIs and REVIs is slightly better than that based on the CIs. In addition, combining indices with different construction principles (red-edge vegetation index combined with visible light vegetation index) can improve the estimation accuracy of the yield to some extent.

**Figure 7:**
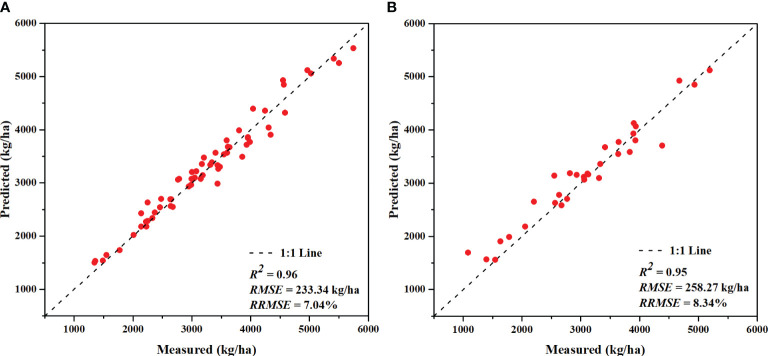
Yield prediction results of the model using the feature combination of the canopy height (CH), fractional vegetation cover (FVC), normalized difference red-edge index (NDVI_RE), and enhanced vegetation index (EVI): **(A)** training set and **(B)** testing set.

Among the three agronomic trait parameters, the combination of the CH and FVC has the best yield prediction (*RRMSE* = 8.93% and *R^2^ =*0.94), which is better than the yield prediction obtained using a single feature and is also better than the results based on the combinations of vegetation indices. Combining the vegetation indices and agronomic trait parameters further improved the yield prediction accuracy. The *RRMSE* of the optimal combination decreased from 10.47–12.65% to 8.34–8.85%, and the *R^2^* increased from 0.88–0.91 to 0.94–0.95. A scatter plot of the yield prediction versus the measured results is shown in **Figure 7**. Therefore, adding agronomic trait parameters to the vegetation indices as feature parameters results in a considerable enhancement of yield prediction accuracy.

### 3.4 Yield prediction across different growth stages

**Figure 8:**
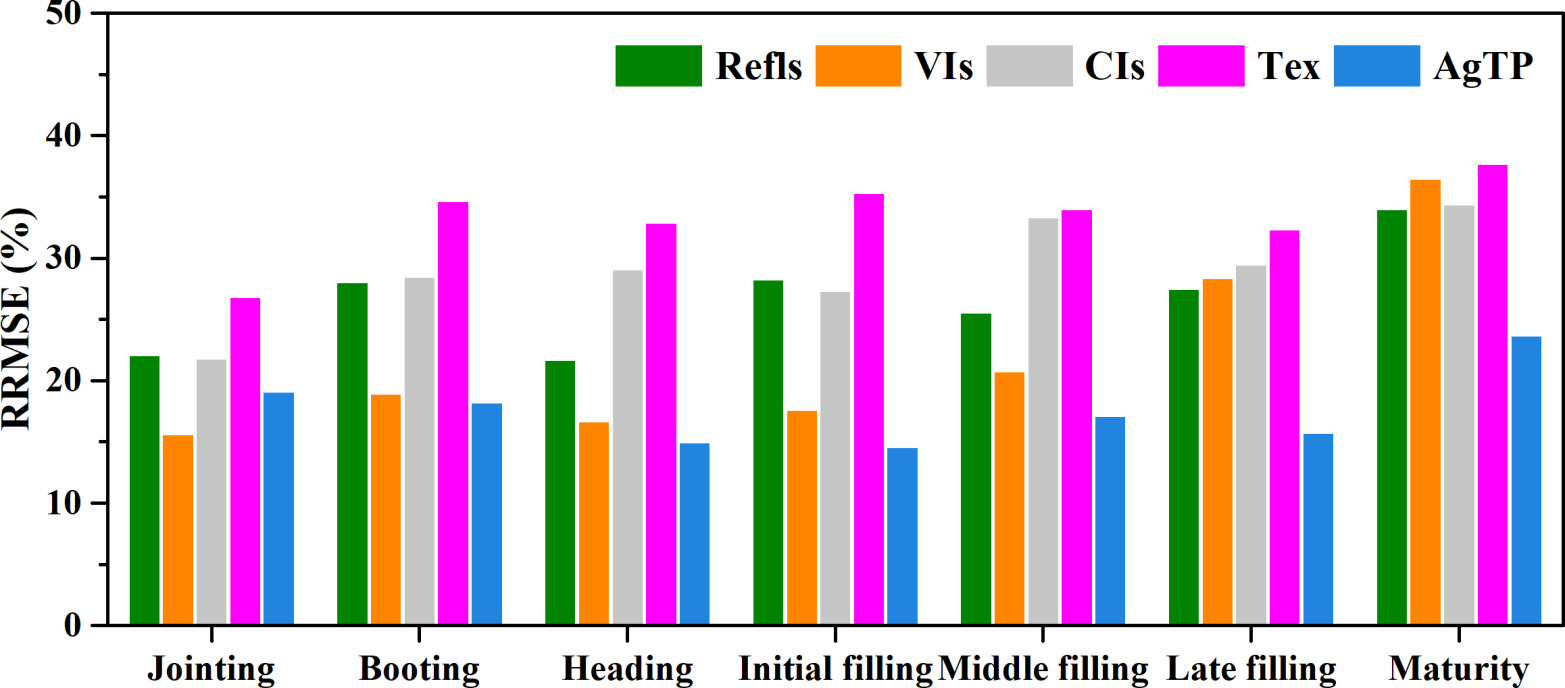
The average *RRMSEs* of the crop yields predicted using a single feature in the different growth stages. Refls, Reflectance; VIs, vegetation indices; CIs, color indices; Tex, texture; AgTP, agronomic trait parameters.

**Figure 9:**
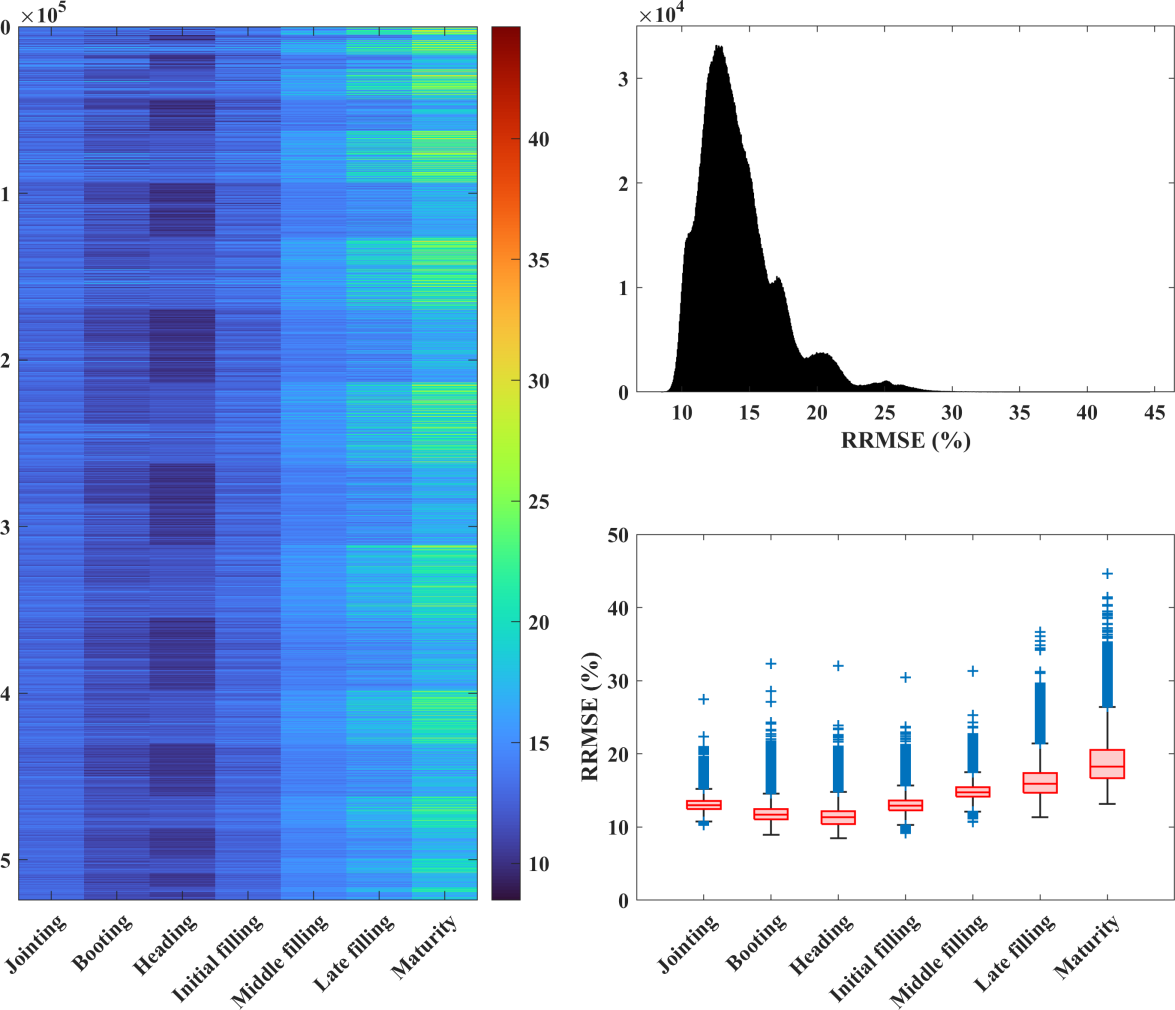
The *RRMSEs* (%) of the crop yields predicted using multiple features in the different growth stages. Left: The colors indicate the *RRMSE* values. The horizontal axis indicates the different growth stages. The vertical axis indicates the different feature combinations of multiple features, and the number of features increases gradually from top to bottom, with a total of 524,287 feature combinations. Upper right: Histogram of the *RRMSE* values; lower right: box charts of the *RRMSE* values for the different growth stages.

The crop growth process includes multiple growth stages, and it is quite important to determine how the features of the growth stages affect the yield prediction. This section mainly exhibits the yield prediction performances in the different growth stages for the use of a single feature and combinations of multiple features. According to the yield prediction results based on a single feature presented in Section 3.2, **Figure 8** shows the average errors in the crop yield predicted using a single feature in the different growth stages. As can be seen from **Figure 8**, the features in the different growth stages make great differences in the yield prediction results. The *RRMSEs* based on a single feature range from 14.6% to 37.7% across different growth stages. Among the different categories of features, the yield errors predicted using the vegetation indices and agronomic trait parameters are relatively small, whereas errors are relatively large for other feature categories. **Figure 9** displays the yield prediction results for the different growth stages using combinations of multiple features (vegetation/color indices and agronomic trait parameters, a total of 19 features). The *RRMSEs* based on combinations of multiple features range from 8.5% to 44.6% across different growth stages. The results also indicate that there are still considerable variations in yield prediction at different growth stages. In general, the prediction accuracies were notably greater in the stages of jointing, booting, heading, and early grain-filling compared to later stages of growth, with the heading stage displaying the highest accuracy in yield prediction (**Figure 9**).

## 4 Discussion

### 4.1 Impact of crop growth stage on yield prediction

In Section 3.4, the study showcased yield predictions across different growth stages, revealing substantial variations in the accuracy of predictions. Notably, the accuracy of yield predictions was found to be superior during the mid-growth phase when compared to the late-growth phase, with the highest accuracy obtained during the heading stage. These findings of this research align with the outcomes of prior studies conducted on wheat (Tanabe et al., 2023) and rice (Wan et al., 2020; Wang et al., 2021). In the later stage of crop growth, the mean and variance of the yield prediction results are large, and the different feature combinations lead to significantly different yield predictions. During the mid-growth stage of crops, the Leaf Area Index (LAI) typically reaches its maximum value, and leaf reflectance in the near-infrared spectrum is at its strongest (Li et al., 2020). Vegetation indices are primarily constructed based on near-infrared radiation. In this stage, vegetation indices exhibit a strong correlation with biomass and yield. Nonetheless, as leaves senescence begin, the capacity of leaves to reflect near-infrared radiation gradually wanes, culminating in the decreased interpretability of vegetation indices for LAI or biomass. Consequently, this progression adversely impacts the accuracy of yield predictions, leading to the lowest accuracy during the maturity stage (Zhou et al., 2017; Tanabe et al., 2023). Similarly, Maimaitijiang et al. (2019) argued that unlike airborne light detection and ranging (LiDAR), photogrammetric point clouds have insufficient penetration ability when the canopy closure is quite high, which may lead to a decrease in the yield prediction accuracy in the later growth stages. Therefore, the features in the jointing, booting, heading, and early grain-filling stages should be preferentially selected for yield prediction, which contributes to a better performance.

### 4.2 Impact of cultivar on yield prediction accuracy

The robustness of the yield prediction models across different cultivars is critical for assessing their application potential (Maimaitijiang et al., 2020; Duan et al., 2021). To evaluate the suitability of the yield prediction models among different cultivars, the data for one cultivar were employed for training, while the data for the other cultivar were utilized for testing. Finally, the mean error of the yield prediction results was calculated. Based on the previous analysis, it can be seen that the yield prediction model with multi-feature fusion is more accurate than that with a single feature. Here, we used multi-feature combinations to analyze the robustness of the yield prediction models among different cultivars. By contrasting **Tables 4** and **5**, it was found that the error of the model, which employed the data for one cultivar to predict the yield of another cultivar, was greater than that of the model trained using the data for both cultivars. The *RRMSE* of the optimal combination of various features increased from 8.34–15.79% to 13.90–19.23%, and the *R^2^* decreased from 0.88–0.95 to 0.81–0.86. The different cultivars of crops have differences in parameters such as phenology, plant height, leaf type, and pigment content. Therefore, the accuracy of the yield prediction models across different cultivars is low. Several recent studies have also reported a decrease in the quality of prediction models for different cultivars (e.g., Rischbeck et al., 2016; Duan et al., 2021). Rischbeck et al. (2016) concluded that models trained using diverse cultivars can significantly improve the yield prediction performance compared to models trained using a single cultivar, which was also concluded in this study. Furthermore, our results support this view.

**Table 5:** Yield prediction results based on various feature combinations and considering cultivar differences.

Types	Feature variables	Number of variables	Number of combinations	Best combination	*RRMSE* (%)	*R^2^*
ComVIs	NDVI, GNDVI, EVI, EVI2, MTVI2, SAVI	6	63	EVI	15.16	0.82
REVIs	NDVI_RE, MSR_RE, CI_RE	3	7	NDVI_RE, CI_RE	19.23	0.81
CIs	NDI, ExG, ExR, ExGR, VARI, GLI, NDYI	7	127	ExG, ExR, NDI, VARI	15.28	0.83
AgTP	CH, FVC, VOL	3	7	CH, FVC, VOL	15.50	0.84
ComVIs+ REVIs+CIs	NDVI, GNDVI, EVI, EVI2, MTVI2, SAVI, NDVI_RE, MSR_RE, CI_RE, NDI, ExG, ExR, ExGR, VARI, GLI, NDYI	16	65535	EVI, NDI	14.32	0.85
ComVIs+AgTP	NDVI, GNDVI, EVI, EVI2, MTVI2, SAVI, CH, FVC, VOL	9	511	CH, EVI, MTVI2	14.51	0.85
REVIs+AgTP	NDVI_RE, MSR_RE, CI_RE, CH, FVC, VOL	6	63	CH, FVC, VOL	15.50	0.84
CIs+AgTP	NDI, ExG, ExR, ExGR, VARI, GLI, NDYI, CH, FVC, VOL	10	1023	CH, ExR, NDI	14.28	0.85
ComVIs+REVIs+ CIs+AgTP	NDVI, GNDVI, EVI, EVI2, MTVI2, SAVI, NDVI_RE, MSR_RE, CI_RE, NDI, ExG, ExR, ExGR, VARI, GLI, NDYI, CH, FVC, VOL	19	524287	CH, EVI, NDI	13.90	0.86

The features of one cultivar were used for training, and the data for another cultivar were used for testing. The values of the error statistics are the average of the two scenarios.

The results of our study indicate that the use of a combination of multi-temporal and multi-features can enhance the yield prediction performance. Therefore, it is quite essential to identify better feature combinations to improve the robustness of the yield prediction models across different cultivars. **Table 5** presents the yield prediction error metrics for various feature combinations across different categories. The results illustrate that the prediction abilities of various feature combinations are different among different cultivars, and the yield prediction accuracy is improved when the agronomic trait parameters are incorporated into the vegetation indices and color indices. This also indicates that the CH, which reflects the vertical growth characteristics of a crop and is one of the important agronomic trait parameters, can better characterize the information about the crop structure and help strengthen the capability of the yield prediction model across cultivars. The combination of the CH, EVI, and NDI indices produced the highest prediction accuracy, with an *RRMSE* of 13.9% and an *R^2^* of 0.86. For the yield prediction models that do not consider cultivars, the REVIs produce larger prediction errors across cultivars.

### 4.3 Importance of using agronomic trait parameters in yield prediction

Through analysis of the previously presented results, we found that when using a single feature for yield prediction, the agronomic trait parameters performed the best overall. Three agronomic trait parameters were used in this study: the CH, FVC, and VOL. Among them, the CH performed best in the yield prediction, followed by the FVC, and finally, the VOL had the weakest performance. The plant CH can reflect the vertical growth characteristics of the crop, can better reflect the information about the crop structure, and can help to improve the yield prediction ability. Since the canopy volume was calculated based on the CH and vegetation coverage, there was an autocorrelation problem, so the performance was not as good as expected.

Furthermore, the models for yield prediction, which incorporated agronomic trait parameters along with spectral features, also demonstrated enhanced accuracy. Existing studies on biomass and yield prediction of other crops (barley, soybean, and corn) have also found that data fusion of spectral and agronomic features can improve the performance (Geipel et al., 2014; Bendig et al., 2015; Maimaitijiang et al., 2019), and this study further supplements related conclusions. The fusion of spectral features and agronomic trait parameters has led to an enhancement in yield prediction accuracy, which can be explained from several perspectives. Firstly, spectral features effectively capture the crop growth status, while multi-temporal spectral features can reflect the entire crop growth and development process (Maimaitijiang et al., 2019; Maimaitijiang et al., 2020; Wan et al., 2020; Tanabe et al., 2023). Secondly, as mentioned earlier, agronomic trait parameters provide valuable insights into crop structural information, particularly vertical growth characteristics that are not easily obtained through spectral features alone. Thirdly, these three agronomic parameters were obtained using UAV-based RGB and MS sensors, which were independent data sources and were not calculated using spectral indices. There was no autocorrelation with the spectral indices, which overcame the inherent asymptotic saturation problem of the spectral features to a certain extent (Maimaitijiang et al., 2017; Maimaitijiang et al., 2020). Therefore, considering the easy availability and cost-effectiveness of obtaining UAV-based agronomic trait parameters, the fusion of spectral indices and agronomic trait parameters has great potential for improving crop yield predictions.

### 4.4 Comparison of yield predictions using RGB and MS images

The features used in this study were all calculated from images acquired by RGB and MS sensors. The VIs and FVC were derived from the MS data, the CIs and CH were derived from the RGB data, and the VOL was calculated based on the CH and FVC, i.e., from a combination of RGB and MS images. Our results confirm that multi-sensor data fusion improves the accuracies of the yield prediction models. While researchers hope to enhance the capacity of the yield prediction, they also expect to achieve this goal at a less cost (e.g., economic cost, time cost, and computational cost). That is, within the range of acceptable accuracy, fewer data and lower costs are more feasible for large-scale applications. Therefore, in this section, we compare the performances of the RGB and MS images in the yield prediction.

**Table 6** shows the yield prediction results obtained using various features obtained from the RGB and MS images. The results indicate that the best yield prediction results were obtained using a combination of the VIs and FVC from the MS sensor, with *RRMSE* = 8.94% and *R^2^ =*0.94. The best yield prediction results from the CIs and CH from the RGB sensor had *RRMSE* = 10.29% and *R^2^ =*0.91. The yield prediction accuracy of the MS-based VIs and FVC was better than that of the RGB-based features. For the RGB-based features, the CH still outperformed the other CIs in terms of the yield prediction, while for the MS-based features, the combination of features involving red-edge indices had a better performance. Red-edge light has a better penetration effect than other visible light bands, is not easily saturated when the vegetation canopy density is high, and is more sensitive to chlorophyll (Dong et al., 2019; Sagan et al., 2021; Zeng et al., 2022).

**Table 6:** Comparison of yield prediction using the RGB and MS images.

Sensors	Types	Feature variables	Number of variables	Number of combinations	Best combination	*RRMSE* (%)	*R^2^*
MS	VIs	NDVI, GNDVI, EVI, EVI2, MTVI2, SAVI, NDVI_RE, MSR_RE, CI_RE	9	511	NDVI_RE, MSR_RE, SAVI	9.72	0.93
	VIs+FVC	NDVI, GNDVI, EVI, EVI2, MTVI2, SAVI, NDVI_RE, MSR_RE, CI_RE, FVC	10	1023	FVC, CI_RE, SAVI	8.94	0.94
RGB	CIs	NDI, ExG, ExR, ExGR, VARI, GLI, NDYI	7	127	ExG, VARI	15.79	0.78
	CIs+CH	NDI, ExG, ExR, ExGR, VARI, GLI, NDYI, CH	8	255	CH	10.29	0.91
MS+RGB	CIs+VIs+CH+FVC+VOL	NDVI, GNDVI, EVI, EVI2, MTVI2, SAVI, NDVI_RE, MSR_RE, CI_RE, NDI, ExG, ExR, ExGR, VARI, GLI, NDYI, CH, FVC, VOL	19	524287	CH, FVC, NDVI_RE, EVI	8.34	0.95

These research results demonstrate that the features that fuse MS and RGB image data have the best yield prediction performance, followed by the MS-based features, and the RGB-based features have the weakest performance. A UAV equipped with an RGB camera is the most common configuration for agricultural RS applications, and this configuration has the advantages of simplicity, convenience, and low cost. Our results show that if the purpose of the research is to understand the crop yield status and the trend from a macroscopic perspective, the RGB-based yield prediction model can fully meet the requirements within the acceptable accuracy range. If the goal is to determine the crop yield more accurately, the use of features obtained from multi-sensor fusion is recommended for yield prediction.

### 4.5 Strengths and limitations of this study and future work

The significant timeliness and operability of UAVs overcome the disadvantages of the spatiotemporal resolution of satellite RS data in precision agricultural applications. UAV-based crop yield prediction has always been an active topic in the field of precision agricultural RS. In this study, RGB and MS images were acquired using a UAV, and crop yield prediction models were constructed based on the RF algorithm and a combination of spectral features and agronomic trait parameters. The results revealed that the model integrating agronomic trait parameters and spectral features enhance the accuracy of the crop yield prediction (**Table 4**; **Figure 7**), and the addition of agronomic trait parameters addressed the issue of reduced prediction capacity across different cultivars to some extent (**Table 5**). In addition, these agronomic trait parameters are easy to obtain at a low cost, so they represent a great potential solution for crop yield prediction at medium and small scales.

Certainly, there were still some limitations in this study. The experiment duration was limited to only one year, and the sample size was relatively small. Multi-year experiments and larger sample sizes would enable a more comprehensive and systematic testing of the crop yield prediction model and feature parameters. Much work remains to be done in the future regarding UAV-based crop yield prediction. First, experiments in different climatic regions need to be conducted to verify the robustness of the yield prediction models across different climatic regions. Experiments involving different crops and different cultivars of the same crop need to be conducted to examine the reliability and suitability of the yield prediction models across crops and cultivars. Second, our research results confirm that multi-data fusion can effectively upgrade the performance of the yield prediction model. The fusion of structural and spectral parameters of crops was adopted in this study. Exploring multi-data fusion, such as thermal infrared, LiDAR, or environmental data, remains a future research focus (Maimaitijiang et al., 2020; Li et al., 2022; Qader et al., 2023). In addition, in terms of machine learning algorithms, previous studies have used deep learning algorithms for yield prediction and have achieved good results (Khaki and Wang, 2019; Khaki et al., 2020; Sagan et al., 2021; Jeong et al., 2022). We also plan to explore the performances of deep learning algorithms in UAV-based yield prediction models in the future.

## 5 Conclusions

Agronomic trait parameters are closely related to crop growth, development, and yield formation. In this study, crop canopy spectral parameters (VIs) and agronomic trait parameters (plant height and coverage) obtained using low-cost UAVs were combined to predict the crop yield. The potential of agronomic trait parameters was also investigated. The main conclusions of this study are as follows:

(1) The agronomic trait parameters and spectral features had strong relationships with the crop yield, while the texture features had relatively weak relationships with the crop yield. Compared with the yield prediction using spectral features, the addition of agronomic trait parameters effectively improved the yield prediction accuracy.
(2) The yield prediction results based on the features in the different growth stages were quite different. In general, the prediction accuracies were noticeably greater in the jointing, booting, heading, and early grain-filling stages as compared to the later growth stages. Early yield predictions were most precise during the heading stage. Multiple growth stages provided a better yield prediction performance than a single stage.
(3) The yield prediction across different cultivars was weaker than that for the same cultivar. However, the combination of crop trait parameters and spectral indices improved the yield prediction among cultivars to some extent.
(4) The features based on MS and RGB fusion had the best performance in terms of the yield prediction, followed by the MS-based features, and the RGB-based features had the weakest performance. It should be noted that the accuracy of the RGB-based yield prediction models also fell within the acceptable accuracy range. Therefore, they meet the requirements for understanding the crop yield status and trends from a macroscopic perspective.

## Data availability statement

The original contributions presented in the study are included in the article/supplementary material. Further inquiries can be directed to the corresponding authors.

## Author contributions

HZ, LS, and HH contributed to conception and design of the study. HZ performed the statistical analysis and wrote the first draft of the manuscript. JY performed the software and programming. WL and DL collected and organized the data. All authors contributed to the article and approved the submitted version.
